# Models of Osteoarthritis: Relevance and New Insights

**DOI:** 10.1007/s00223-020-00670-x

**Published:** 2020-02-15

**Authors:** Hasmik Jasmine Samvelyan, David Hughes, Craig Stevens, Katherine Ann Staines

**Affiliations:** School of Applied Sciences, Edinburgh Napier University, Sighthill Campus, Edinburgh, UK

**Keywords:** Osteoarthritis, In vitro models, 3D cell culture, Ex vivo models, In vivo models

## Abstract

Osteoarthritis (OA) is a progressive and disabling musculoskeletal disease affecting millions of people and resulting in major healthcare costs worldwide. It is the most common form of arthritis, characterised by degradation of the articular cartilage, formation of osteophytes, subchondral sclerosis, synovial inflammation and ultimate loss of joint function. Understanding the pathogenesis of OA and its multifactorial aetiology will lead to the development of effective treatments, which are currently lacking. Two-dimensional (2D) in vitro tissue models of OA allow affordable, high-throughput analysis and stringent control over specific variables. However, they are linear in fashion and are not representative of physiological conditions. Recent in vitro studies have adopted three-dimensional (3D) tissue models of OA, which retain the advantages of 2D models and are able to mimic physiological conditions, thereby allowing investigation of additional variables including interactions between the cells and their surrounding extracellular matrix. Numerous spontaneous and induced animal models are used to reproduce the onset and monitor the progression of OA based on the aetiology under investigation. This therefore allows elucidation of the pathogenesis of OA and will ultimately enable the development of novel and specific therapeutic interventions. This review summarises the current understanding of in vitro and in vivo OA models in the context of disease pathophysiology, classification and relevance, thus providing new insights and directions for OA research.

## Introduction

Osteoarthritis (OA), a common, chronic joint disorder, is a leading cause of disability and healthcare costs worldwide. The prevalence of OA varies depending on age, gender, race and geographical location. It is estimated that worldwide 10% of men and 18% of women aged over 60 years have symptomatic OA [1]. OA affects over 8 million people in the UK alone [2] and, therefore, the predicted increase in the ageing population will only result in a greater occurrence of the disease.

OA is a complex degenerative disease for which the cellular and molecular mechanisms of initiation and progression are incompletely understood. OA results in the progressive loss of articular cartilage (AC) and thickening of the underlying subchondral bone (SCB), and is perpetuated by abnormal cartilage restoration and bone remodelling [3]. As OA progresses, the condition is characterised by joint space narrowing [4], as well as the formation of bone marrow lesions, cysts [5] and tidemark duplication [6]. Additionally, the calcified cartilage region is found to increase in volume and is subject to vascular penetration which eventually reaches the non-calcified AC. Outside of the osteochondral tissues, the synovium surrounding these tissues is frequently inflamed, undergoing fibrosis and vascularisation [7]. OA clinical symptoms present as pain, stiffness, swelling and a decreased range of motion [8]. Various risk factors exist for OA including age, obesity, and physical trauma [9], as well as genetic predisposition [10]. At present, there is a paucity of non-invasive therapies available to patients and, therefore, there is a pressing need to develop novel targeted and effective disease-modifying treatments.

The diversity of risk factors that influence the AC and SCB in OA, together with the poor transition from in vitro to in vivo studies necessitates a validated in vitro model to investigate disease pathology [11]. Typically, in vitro cell models are two-dimensional (2D) and do not faithfully model physiological conditions, producing a limited extracellular matrix (ECM)-like environment that results in altered cell morphology [12]. Novel three-dimensional (3D) models of OA retain the benefits of 2D models whilst additionally providing a customisable environment which is more comparable to physiological conditions [13, 14]. Thus, 3D models allow the analysis of cell–cell interactions, cell-ECM interactions and cellular responses to pathological stimuli.

Several animal models have been developed to study the pathogenesis of OA and the efficacy of new diagnostic tools and therapeutic interventions. However, due to the heterogeneity of the disease there is no single animal model that reflects the onset and progression of OA in humans [15]. Whilst there are similarities in the disease process between humans and animals, no single animal model is sufficient to study all the degenerative features of OA, thus model selection is based on the aetiology under investigation and the intended purpose. This review summarises the benefits and the limitations of current 2D and 3D in vitro models, and the classification and relevance of in vivo models to human pathogenesis, thereby providing future directions into OA research.

## In Vitro Models of OA

A variety of in vitro models have been used to study the pathogenesis of OA at both tissue and cellular levels; however, no single model has proven to be the gold standard for OA research [16]. To date, publications using 2D cell culture primarily consider the chondrocyte as the “model” for OA, with little consideration of the role of other cells relevant to the articular joint such as SCB osteoblasts and osteocytes [17]. Therefore, to create a robust and reliable model of OA, the variety of articular joint tissues must be considered and investigated as a biological unit. Furthermore, in vitro and in vivo models often produce conflicting results. Thus, there is a need for an in vitro model that generates results consistent with in vivo studies, and that overcomes the limitations of the current 2D and 3D models. Below, we have summarised in vitro models currently used for the investigation of OA (Table 1).

**Table 1:** Advantages and disadvantages of in vitro models of osteoarthritis

Model	Advantages	Disadvantages	References
Monolayer	Inexpensive, high throughput, exposes cells to equal volumes of nutrients and growth factors, allows for the preliminary investigation of the mechanisms underlying disease pathology, as well as assessment of the effects of specific compounds on cell phenotype	Can induce cell de-differentiation, alters cell morphology, cells can become polarised altering their phenotype, has limited potential for investigation into cell–cell and cell-ECM interactions, and has restricted capacity for investigation of loading on tissues	[12, 25, 26, 29]
Co-culture	Facilitates investigation into cell–cell and cell-ECM interactions, allows investigation of the effects of specific compounds on cell phenotypes and interactions, and facilitates investigation into loading regimens on the OA phenotype	Requires further refinement of culture conditions, can be costly, cells may de-differentiate depending on co-culture system (i.e. inclusion of a 2D component such as transwell plates)	[31, 32, 36]
Explant	Considers tissues as a whole, facilitates investigation into cell–cell and cell-ECM interactions, and has potential for co-culture	Tissue sources are finite, explants are more financially costly than monolayer models, and cells at surgical edge of tissues may die and influence analysis	[33–35]
Scaffold and scaffold-free systems	Maintains cell-differentiated phenotype. Can be highly modifiable. Allows investigation of the effects of stimulatory molecules such as cytokines on cells. May be included in a co-culture system, facilitates investigation into cell–cell and cell-ECM interactions under disease phenotype. Has potential to allow for investigating the effects of loading on cells in vitro	Can be costly, 3D culture is still in infancy, cell proliferation is decreased, a broad range of model systems and raw materials exist which can influence cell activity necessitating additional optimization for in vitro model development	[38, 45, 47, 49, 52, 58, 61, 64]

### 2D Cell Models

#### Monolayer

2D monolayer models are inexpensive, permit the use of a single source of cells for multiple experimental treatments, and allow for rigorously controlled investigational conditions (Table 1). Typically, 2D monolayer models involve the culturing of either primary cells or immortalised cell lines on a flat surface in polystyrene culture flasks, which exposes the cultured cells to an equal volume of the surrounding media containing the various nutrients and growth factors essential for cell development and proliferation [18, 19]. 2D in vitro models have routinely allowed for the screening of chondroprotective compounds to attenuate the catabolic factors involved in AC degradation. Usage of primary cells including osteoblasts and chondrocytes in 2D models provides a system that is associated with higher secretion of ECM and a phenotype more akin that found in vivo for the respective cell lineage. However, it is of note that primary cell culture is substantially more expensive than the use of cell lines, with primary cell source material being difficult to obtain and limited in terms of how often they can be subcultured before they undergo de-differentiation and lose their distinct phenotype. The usage of cell lines provides a much less limited source of cell material however due to their immortalised nature, these cells have evaded cellular senescence and are associated with alterations in expression of markers typically associated with their non-immortalised counterpart. This can be exemplified by immortalised chondrocytes which display a marked reduction in the secretion of ECM components compared to primary cells, thus reducing their reliability as true cell models. 2D in vitro models have routinely used for cytokine stimulation such as IL-1β to induce an OA phenotype which has allowed for the screening of chondroprotective compounds to attenuate the catabolic factors involved in AC degradation (e.g. IL-1β, TNF-α NO, PGE_2_, COX-2, MMP3, MMP13, ADAMTS-4 and ADAMTS-5) [12, 20–24]. Manipulation of 2D culture can also allow for investigation of individual signalling pathways. For example, recent interrogation of the Wnt/β-catenin signalling pathway with a novel inhibitor highlighted the critical role of the Wnt/β-catenin pathway in chondrogenesis and chondroprotection [25]. Additionally, monolayer cultures are easily transfected to manipulate gene and protein expression. For example, a proteomics study conducted on IL-1β/TNF-α stimulated human primary chondrocytes that were transfected with microRNAs (miRNA) identified proteins of the complement cascade, mediators of the NF-κB pathway and several regulators of autophagy that may be important in the pathology of OA [26].

Despite the benefits of 2D cell culture, there are numerous drawbacks that are inherent in the nature of the system itself (Table 1). When using primary cells in 2D culture such as primary chondrocytes, little consideration is given as to the location from which these cells are derived, be it from articular, costal or fibrillar cartilage, or a specific layer of the cartilaginous matrix. Notably, 2D cell culture results in altered cell morphology as cells are forced to grow in a planar environment where nutrient and oxygen gradients are non-existent [27]. This can result in cellular polarisation due to limited connections with surrounding cells. Polarisation is known to alter cell mechanotransduction; directly impacting cell signalling and, therefore the phenotype of the cell [28]. Chondrocyte cultures are no exception to this as they have been found previously to de-differentiate and adopt an elongated fibroblast-like shape that has been associated with an altered genetic profile, including a reduction in the expression of aggrecan and other matrix specific genes [29]. Additionally, prolonged monolayer culture of chondrocytes results in the increased expression of *Col1a1* mRNA, thus, signifying de-differentiation [30]. Furthermore, incongruous results are often found between in vivo and in vitro studies. Also, there is a lack of support for investigation into mechanosensory stimulation in monolayer cell culture systems, which is also limited in its use in investigating cell–cell interactions and cell-ECM interactions. Therefore, to create a robust and reliable model of OA, the variety of articular joint tissues must be considered as a biological unit.

#### 2D Co-culture

2D co-culture models allow the investigation of cell–cell interactions in a shared environment. Like monolayer models, these can be used to investigate multiple experimental treatments at once and have the potential to generate extensive data on pathological mechanisms (Table 1). Transwell plate models may be used which involves seeding cells in the lower chamber of multi-well plates, with additional cells being seeded in transwell plates suspended above each well. This allows for investigation of cell–cell communication via the secretion of soluble factors into the surrounding media [31, 32]. Using this method, it has been shown that when chondrocytes and synoviocytes are co-cultured alongside adipose-derived mesenchymal stem cells, their expression of pro-inflammatory cytokines including IL-1β, IL-6, IL-8 and TNF-α significantly decreases [31]. A similar method revealed that chondrocytes cultured in the presence of adipose-derived stem cells have higher viability following TNF-α challenge, thus highlighting a paracrine chondroprotective role for adipose-derived stem cells [32]. As detailed, these experiments allow for the exploration of a variety of factors on OA development. However, it should be noted that many of the limitations associated with 2D monolayer culture also exist in 2D co-culture, including altered cell morphology and an inability to investigate direct cell–cell or cell-ECM contact.

### 3D Cell Models

Due to the limitations of 2D cell models, development of a more robust and reliable in vitro culture system to investigate OA is required. Specifically, a model is required that is reproducible, financially viable, and that allows the investigation of cell–cell and cell-ECM interactions in disease pathology, as OA should be considered a disease of the whole joint as opposed to one particular aberrant cell type. 3D cell models may therefore present a good alternative to 2D culture. 3D models for the in vitro analysis of SCB and AC currently exist in a variety of forms, including explants and scaffold-based or scaffold-free systems, each with their own advantages and disadvantages (Table 1). However, a common feature of these 3D models that makes them suitable for a variety of applications is their ability to maintain the phenotype of cells such as AC chondrocytes.

#### Explants

Explant models are derived directly from in vivo tissue and maintain cells in their 3D surroundings, and both animal (e.g. murine femoral heads) and human (e.g. AC, or whole osteochondral plugs) explant systems are used within the OA field. These models still allow for the experimental manipulation provided by in vitro culture, with experimental evidence suggesting tissue viability is maintained [33]. Explant models have certain benefits such as enabling the investigation of compressive overload on AC, providing insight into the impact of cartilage loading in disease progression; a facet of OA that cannot be investigated by monolayer models [34]. Comparable to monolayer culture, explants may undergo multiple treatments in vitro allowing for the investigation of a variety of factors in a controlled environment. Unlike monolayer culture, osteochondral explant models may be more readily used to investigate the relationship between AC and the underlying SCB tissue. Osteochondral explants challenged with IL-1β have been found to express a significantly lower level of MMP13 than cartilage explants alone; results from this study also indicate that osteochondral explant expression of alkaline phosphatase may differ from chondrocyte explants [34]. Additionally, cartilage explants challenged with IL-1β produced an elevated level of TNF-α compared to controls, whereas osteochondral explant TNF-α secretion was unchanged, suggesting the presence of bone or synovium may reduce TNF-α expression and highlighting the need to consider all joint tissues in the analysis of OA pathophysiology [35]. In agreement, bone explants have been found to secrete increased levels of pro-Col-I, IL-6 and MCP-1 compared to osteochondral explants when challenged with lipopolysaccharide, suggesting that different cells within the osteochondral tissues can attenuate one another [33]. Furthermore, Haltmayer et al. [36] detailed a co-culture model in which horse osteochondral plugs and synovium membrane explants were cultured in vitro and stimulated using IL-1β and TNF-α. This study showed that the osteochondral-synovium co-culture enabled upregulation of MMP1 expression, subsequently attenuating genetic expression of MMP3, MMP13, IL-6 expression, as well as increasing genetic expression of ECM products such as collagen type II.

Clearly, explant models provide benefits over monolayer culture, particularly regarding interactions between tissues; however, there are still shortcomings that must be addressed when using these models. For example, cells at the surgical edge may die when tissue is removed from specimens, and tissue from any single same biological source is finite; with different sources potentially eliciting different responses [35]. Importantly, explanted tissues are cultured in artificial settings which limit investigation of mechanical loading effects or angiogenic effects following surgical resection [33]. Additionally, cells derived from explant outgrowths are susceptible to the de-differentiation and morphology changes observed over time in 2D culture, and there is still a requirement for use of culture media which may contain components that have undesirable effects on the tissues [33].

#### Scaffold-Based Systems

3D tissue scaffolds provide a platform in which biochemistry, matrix elasticity and micro-architecture can be altered [37]; this is important as polarity, pore size and pore interconnectivity affect cell fate and the ability of cells to secrete ECM products. Additionally, biologic hydrogels have the ability to support chondrocyte proliferation and ECM production, as well as osteoblast growth and mineralisation, providing a potential model for investigating activity at the osteochondral interface [37–39].

Biologic hydrogels represent a scaffold-based system that are derived from natural resources, have a large water component and are used due to their similarities to ECM modifiability, bioactivity, biodegradability, porosity, biocompatibility and low immunogenicity. Biologic hydrogels derived from materials such as alginate, gelatin, chitosan and hyaluronan promote chondrocyte viability and proliferation, as well as collagen type II, aggrecan and Sox9 expression, markers which normally diminish in monolayer culture [40–42]. Additionally, biologic hydrogels have the ability to support chondrocyte proliferation and ECM production, as well as osteoblast growth and mineralisation, providing a potential model for investigating activity at the osteochondral interface an area important in OA pathology [43]. Remarkably, certain biologic hydrogels can be manufactured through 3D printing, increasing availability and reducing the need for fabrication and crosslinking with potentially toxic reagents and hazardous processes [44]. Recent work on developing an *in* vitro 3D model of OA by Galuzzi et al. [45] has shown that nasal chondrocytes encapsulated within alginate beads are able to produce increasing levels of Glycosaminoglycans (GAG); however, culture in these beads had no effect on AC chondrocyte GAG secretion, highlighting a potential limitation for their use as a model of OA. Unfortunately, biologic hydrogels can suffer from variation in batch to batch manufacturing, as with explants, single biological sources can vary which can impact the properties of the gel. These must be considered when choosing a biologic hydrogel culture system.

Synthetic hydrogels are prevalent in 3D tissue culture, and like biological hydrogels they possess desirable features for tissue culture [46, 47]. Importantly, synthetic hydrogels do not originate from a finite source meaning variability between manufactured products is reduced [48]. Synthetic hydrogels have been shown to facilitate chondrogenesis and increased expression of key chondrocytic markers such as collagen type II, and non-collagenous proteins such as osteocalcin, compared to 2D controls [49, 50]. Synthetic hydrogels can also be finely tuned via chemical modification, such as the inclusion of chondrogenic molecules which are delivered to hydrogel embedded cells such as biotinylated TGF-β3 which promotes chondrogenesis, as well as collagen type II and GAG expression [51]. Stüdle et al. describe a model in which bone marrow stem cells (BMSCs) seeded in Poly (ethylene glycol) (PEG) hydrogels are layered with non-functionalised PEG-seeded nasal chondrocytes. This 3D construct results in a calcified bottom layer of BMSCs and a cartilaginous top layer of chondrocytes producing a 3D co-culture model interface of SCB and AC-like tissues which could be adapted for investigation into OA pathology [51]. Similarly, a hydrogel co-culture system has been produced [52] using transwell plates to elucidate the relationship between chondrocytes and macrophages in OA. Research has also shown synthetic hydrogels such as those derived from PEG dimethacrylate are able to be integrated into a mechanical loading system which was able to direct human mesenchymal stem cell differentiation into AC, calcified cartilage and SCB tissues [47]. Preliminary data therefore supports the potential of a synthetic 3D hydrogel co-culture system that includes multiple cell types involved in OA to investigate cell–cell interactions at a molecular level in the context of OA development.

Hydrogels thus provide materials that have a variety of suitable characteristics for in vitro modelling; however, certain disadvantageous features exist. In vitro modelling must consider the composite parts of these hydrogels as they are comprised of materials that are dissimilar to those found in the natural ECM and therefore may alter cell behaviour. Moreover, these hydrogels contain a large water component, which has implications for the structural integrity of the gel as an anchoring substrate and while this may be suitable for mimicking certain in vivo environments such as AC, it may be unsuitable for others such as SCB which has very little ECM water content in vivo.

Pre-fabricated scaffold, comprised of biodegradable polymers, are favoured for their biocompatibility, allowing for easy integration into biological systems as exemplified using PCL [poly(ɛ-caprolactone)] in surgical sutures and implants. Pre-fabricated scaffolds also possess a low melting point, providing desirable thermoplastic properties which facilitate 3D printing. Additionally, the viscoelastic properties of these scaffolds provide benefits for cell culture as substrate stiffness can determine cell growth [53, 54]. However, it has been noted that polymers such as PCL are non-osteoinductive and therefore their use in the culture of bone tissues is considered limited, limiting their potential to be used to investigate an osteoblastic OA phenotype [55]. To remedy this, recent research has taken to altering PCL scaffold biochemistry and fabrication methods. Indeed, osteoinductive and mechanically supportive molecules such as hydroxyapatite (HPA) and Poly(propylene fumarate) (PPF) have been included to facilitate bone growth [38]. PCL/HPA/PPF scaffolds were found to boost BMSC osteoinduction increasing levels of calcium deposition and *Runx2* expression, a marker of osteoblast differentiation, and were particularly noncytotoxic [38]. In consideration of improving manufacturing methods, Brennan et al. [56] developed a novel jet-spraying technique as an alternative to the commercially accepted electrospinning technique. Electrospinning produces nanofibre scaffolds that closely resemble the native bone ECM; however, cell infiltration is a common problem with these scaffolds. Jet-spraying manufacturing produced scaffolds that boost alkaline phosphatase levels and calcium deposition whilst maintaining collagen production at similar levels to commercially available electrospun scaffolds – this is due to jet-spraying producing scaffolds with smaller pore diameters and a greater variety of fibre thickness within the scaffold. Notably, these scaffolds resulted in higher osteogenesis than their 2D counterparts [56].

Pre-fabricated scaffolds also include microcarriers which do not possess the mesh-like physical properties expected of a scaffold, but instead anchor cells on their surface providing 3D support [57]. Thus far, microcarriers have shown desirable chondrogenic qualities. Galuzzi et al. [45] described the development of a silk/alginate microcarrier model in which silk anchors human nasal chondrocytes to the surface of the silk/alginate manufactured beads avoiding encapsulation and negating issues associated with cell infiltration. These cells retained a chondrocytic phenotype via expression of markers such as collagen type II and were metabolically active even after cryopreservation [45]. Microcarriers have also been shown to support biochemical modification [58]. A microcarrier consisting of PGLA (poly(d,l-lactide–co-glycolide acid) coated with fibronectin and poly-d-Lysine to promote cell adhesion, and loaded with TGF-β was shown to upregulate chondrogenic markers, whilst downregulating osteogenic proteins [58]. This highlights the ability of 3D culture models to be tailored to guide stem cells to a specific lineage giving more control over differentiation than can be found in 2D models.

By showing chondrogenic and osteogenic potential that can also facilitate cell–cell and cell-ECM interaction, scaffold-based systems therefore offer great potential as in vitro systems to model OA. Notably, cell proliferation is typically found to be slower in scaffold-based systems [59], as is cell migration [60]. However, research involving these models is still in relative infancy compared to 2D in vitro models. Further work is needed to establish the implications of these characteristics on model development as well as the robustness and reliability of these systems.

#### Scaffold-Free Systems

Pellet culture provides an alternative to typical 3D culture that carries less of a financial burden than other 3D systems such as hydrogels and scaffolds. Typically, pellet culture involves maintaining centrifuged cell pellets in conical tubes, or multi-well plates, in such a manner that the cells are clumped together, adding a 3D aspect to the culture system. De-differentiated human AC chondrocytes cultured as a pellet have shown increased expression of chondrogenic SOX9 as well as *Col2a1* and aggrecan mRNAs [61]. Additionally, pellet cultured chondrocytes exhibit reduced expression of hypertrophy markers such as collagen type X, as well as a reduction in calcification markers such as *Runx2* and alkaline phosphatase. Pellet culture also induces collagen type II expression to a higher level than both 3D alginate bead culture and monolayer culture; however, expression of collagen type X and *Runx2* protein did not differ between monolayer, pellet or alginate bead culture [61].

In contrast to the benefits described, pellet culture has inherent systematic disadvantages that must be considered when using this in vitro culture method, particularly for OA. Cells cultured in a pellet show a large reduction in proliferative capacity, whilst cells in the centre of the pellet may be deprived of nutrients and oxygen; providing an environment for maintaining non-hypertrophic chondrocytes for in vitro analysis [62]. However, these conditions can cause apoptosis; and more recent research has provided an alternative chondrogenic model using transwell plates. Whilst these hypoxic and low nutrient conditions may be suitable for chondrogenesis, it has been shown that hypoxia can reduce osteogenesis [63]. If an in vitro model of OA is to be fully comprehensive, it must suitably support the variety of cell types that are found within an articular joint including chondrocytes and osteoblasts.

Hanging drop cell culture involves pelleting cells and culturing them in an inverted fashion against a 2D surface, allowing gravity to help maintain the cells in suspension reducing the chance of polarisation. In vitro culture using the hanging drop technique has been shown to promote an in vivo-like rounded morphology accompanied by increased levels of *Sox9* mRNA compared to both monolayer and typical pellet culture method. Additionally, hanging drop culture has been shown to induce upregulation of proteoglycan 4 (Prg4) mRNA and its associated protein lubricin—which is found in healthy AC joint space, providing lubrication [64]. Hanging drop models present similar advantages as pellet culture systems, but notably share the same disadvantages and therefore further research is required to determine their suitability as a potential adaptable model for the in vitro investigation into OA.

## In Vivo Models of OA

Clinical and preclinical studies have proven to be indispensable tools to study the pathogenesis and progression of OA. However, the chronic nature of the disease, variability in the onset of symptoms and rate of progression in humans present challenges for clinical studies [65]. Further, it is difficult to obtain human samples during early stages of disease because patients mainly present in the clinic after OA has developed. OA research currently relies on in vivo models in which disease susceptibility and progression can be easily defined, allowing identification of the aetiological factors that lead to OA. Thus the knowledge gained from these preclinical models could be essential in developing early therapeutic interventions.

At least 18 animal models have been developed to study pathophysiological features and pathogenesis of OA [66]. The advantages of smaller model organisms such as zebrafish, rodents (mice and rats), guinea pigs and rabbits include relatively low cost, ease of handling and maintenance. As a result, they are often used as the first models for developing therapeutic interventions and initial drug-screening studies [67, 68].

Large model organisms including dogs, goats, sheep and horses are used to study the pathological process of OA and develop more clinically relevant features due to their striking anatomic similarities to humans including joint size and AC thickness. Other advantages of large animal models include prevalence of naturally occurring primary and secondary OA, feasibility of arthroscopic intervention and diagnostic imaging such as MRI [69]. These models are required to test the efficacy of drugs prior to the clinical trials and approval of therapeutic interventions by the regulatory authorities [70]. The disadvantages of large animal models are mainly related to cost, handling challenges, longer time to age, slower progression to OA and ethical considerations [67].

Based on the disease aetiology, OA can be classified into two main types: primary or idiopathic OA and secondary OA. Primary (idiopathic) OA is described as naturally occurring OA affecting one joint (localised) or three or more joints (generalised), whereas secondary OA is associated with a variety of causes and risk factors leading to disease including trauma, metabolic bone and congenital disorders [71]. Due to its heterogeneous nature, OA can be further categorised into clinical phenotypes including post-traumatic, metabolic, ageing, genetic and pain phenotypes [72]. Current animal models for OA can also be broadly classified into these subtypes, and it is imperative that these classifications are considered at the time of model selection and subsequently when interpreting results (Table 2).

**Table 2:** Advantages and disadvantages of in vivo models of osteoarthritis

Model	Advantages	Disadvantages	References
Naturally occurring models	Spontaneous models, no need for intervention, used to study pathogenesis of naturally occurring OA, variable in OA manifestation such as in human OA	Time-consuming due to slow progression of OA, high cost	[74, 82, 89, 90]
Genetically modified models	Spontaneous models, easy to produce, used to study the contribution of specific genes in OA and develop disease-modifying treatments	High cost, production of additional cartilage abnormalities and potential lethal gene deletions	[68, 93]
Surgically induced models	Rapid progression of OA and therefore short study timeframe, reproducible, induces post-traumatic OA, allows the study of various lesions/stages of disease and assess therapeutic efficacy of agents for OA treatment	Inappropriate for studies of degenerative OA since generated by traumatic invasive intervention	[95–100]
Chemically induced models	Most rapidly progressing OA, easy to implement, relatively less invasive than surgically induced models, reproducible, useful for short-term studies, allows the study of various lesions/stages of disease and assess therapeutic efficacy of pain-alleviating agents for OA treatment	Rapid and widespread changes generated by invasive intervention, poor correlation with the pathogenesis of human OA	[104, 105]
Non-invasive models	Non-invasive, severity of the lesions can be adjusted, low risk of infection, reproducible, allows the study of early OA changes after acute or chronic overuse injuries of joints and the effects of early therapeutic intervention	Equipment is not commonly available, several loading cycles and episodes are needed to induce severe OA changes, still in the early stages of understanding its application	[106–108, 115]

### In Vivo Models of Primary OA

Spontaneous animal models of OA are commonly used to study primary OA and subcategorised into naturally occurring and genetically modified models. These models exhibit slow progression of the disease which imitates natural progression of the human primary OA, and thus, are time-consuming but pathophysiologically are closely related to the human disease (Table 2) [15, 70].

#### Naturally Occurring Models

Naturally occurring models of OA include mice, certain strains of guinea pigs, rabbits, dogs and horses. Different mouse strains exhibit different OA vulnerability. Particular strains including STR/ort and C57BL/6 are considered predisposed to developing spontaneous idiopathic OA. Other strains, notably the CBA mice are considered to have a resistance to the development of spontaneous OA and, therefore, the lack of overt OA makes them effective controls for these studies [73]. The STR/ort mouse is a well-recognised model of naturally occurring OA with disease pathology starting early in life and showing similar characteristics to human primary OA. The transgenic STR/ort mouse, directly derived from STR/1 N strain, has featured in over 80 studies of OA [74]. The first studies reported increasing incidence and severity of OA from 18 weeks of age and described a greater incidence of osteoarthritic knee, elbow, ankle and temporomandibular joint pathology, specifically in male mice [75, 76]. In STR/ort mice AC undergoes structural deterioration similar to human OA due to early changes in AC matrix integrity and composition [77], a transient chondrocyte phenotype and altered function [78], dysregulation of cell signalling pathways such as TGF-β and Wnt [79, 80] and increased oxidative stress [81].

The albino Dunkin-Hartley or Hartley guinea pigs are widely used to study naturally occurring OA, primarily due to the histopathological similarities to human primary OA and their rapidity of growth to skeletal maturity compared to other larger animal spontaneous models of OA [82, 83]. Other advantages include the size of the joints allowing sufficient tissue and synovial fluid collection for relevant downstream analyses, and the ability to study joint inflammation, as well as ease of handling of the species [84]. Additionally, the Hartley guinea pig is a useful model to study OA-associated pain and evaluate feasibility of novel therapies, including nociception inhibitors, human mesenchymal stem cells, gene therapy and RNA interference [85–87].

Previous studies showed radiographic evidence of naturally occurring OA in 50% of rabbits over 6 years of age and more than 70% of rabbits older than 9 years of age [88]. Rabbits are used for bioengineering experiments to develop treatments for diseased cartilage [89]. Dogs and horses are also beneficial as translational models of naturally occurring OA for preclinical studies to develop novel and effective therapeutic interventions [90]. The front knee of the horse with two layers of carpal bones is analogous to the human wrist. The metacarpophalangeal and carpal joints of the horse exhibit close fitting of articular surfaces and are most susceptible to primary OA. The articular surfaces develop erosions and wear lines due to osteochondral defects and fragmentation in these joints. The SCB sclerosis and subsequent focal osteonecrosis occur as disease progresses [91, 92], thus, this model has been used to study AC repair, osteochondral defects and bone remodelling that leads to osteophyte formation.

#### Genetically Modified Models

Genetically modified models including knockout and knock-in animal models are useful to determine the genetic factors involved in OA pathogenesis, including the function of specific genes associated with AC degradation, SCB remodelling and inflammation [69, 70]. Thus, specific genes or gene products involved in the protection of premature AC degradation can be exploited to develop disease-modifying treatments. The zebrafish model displays phenotypic characteristics of OA including reduced joint mobility, loss of AC and formation of bony spurs. It has been used for functional studies on OA susceptibility genes that play a role in disease [68]. The entire mouse genome has been sequenced; thus, it is relatively easy to produce genetically modified mouse models of OA and these have been used extensively to study genotype–phenotype relationships. These mice are compared to their wild-type counterparts to evaluate spontaneous development of the disease, or are used in combination with surgically or chemically induced models of OA, as discussed in the next section [93].

### In Vivo Models of Secondary OA

Secondary OA associated with injury, insult or trauma to the affected joint (post-traumatic OA) is the most widely studied subtype of OA [94]. Secondary OA is investigated by inducing direct or indirect injuries to the joints, using invasive and non-invasive models of OA (Table 2).

#### Invasive Models

Invasive models are largely used to study the pathogenesis of post-traumatic OA and to assess the therapeutic efficacy of drugs/potential therapeutic agents for the disease. The advantages of invasive models include rapidly progressing OA and, therefore shorter study timeframes, induction of severe lesions, reproducibility and relatively low cost compared to the spontaneous animal models. Nonetheless, these models are inappropriate for the pathogenetic studies of naturally occurring primary degenerative OA. Invasive models of OA include surgically induced and chemically induced models. Invasive surgical procedures induce OA by disrupting joint biomechanics, producing inflammation, instability and altering load-bearing of the joints, whereas relatively less invasive intra-articular injection of chemical agents and inflammatory compounds including monosodium iodoacetate (MIA), papain, quinolone and collagenase alter joint homeostasis and lead to the histological and morphological destructions of its structures [15].

Transection of the anterior cruciate ligament (ACL) is one of the most frequently used surgical methods for studying OA in vivo. The ACL injury causes destabilisation of the joint, leading to post-traumatic OA and imitating AC degradation. The slow development of OA lesions makes this surgical model advantageous for pharmaceutical studies. In mice and rats, transection of ACL is performed either alone or in combination with transection of the posterior cruciate ligament (PCL) or medial or/and lateral collateral ligaments, as well as meniscectomy. Such combinations allow researchers to study the grades and various stages of OA development [95]. Animal models including goats, sheep and cows are used for transection of ACL due to the large size of their stifle and, therefore, easy replication of the surgical procedure and anatomical similarities to the human knee [15].

Destabilization of the medial meniscus (DMM) is a well-established and widely used surgically induced model of OA. This model can be used for target validation studies using genetically modified animals and evaluation of the pathophysiological roles of various molecules and enzymes in OA in vivo. The DMM murine model involves sectioning of the medial meniscotibial ligament (MMTL), which anchors the medial meniscus to the tibial plateau, giving destabilisation of the medial meniscus. Following DMM, medial displacement of the medial meniscus in a mouse knee provides a smaller area to transmit the weight-bearing forces and leads to an increased local mechanical stress [96]. DMM model induces OA with great ease, provides extremely good reproducibility and slow progression of the disease, thus resembling slowly progressive human OA and allowing evaluation of disease-modifying OA drugs. Most DMM studies are conducted on young adult animals; however, when DMM is performed on aged animals the ensuing OA is more severe due to the age-related changes in a basal pattern of gene expression in joint tissues. Furthermore, induced OA may be more progressive in male than in female mice following DMM [97]. Thus, animal age and gender should be taken into consideration when modelling molecular mechanisms to define therapeutic targets for OA [98].

In mice, total medial meniscectomy in combination with transection of the medial cruciate ligament (MCL) engenders osteoarthritic changes around 8 weeks after surgery. In rats, meniscectomy generates AC defects 1 week and changes in SCB 2 weeks after surgery. While in humans, surgical removal of a meniscus following knee injury represents a significant risk factor for radiographic OA after 20 years [99]. This procedure can also be performed in rabbits, dogs, sheep and monkeys. The site for the surgery varies depending on the load-bearing capacity of each animal, either on its medial or lateral menisci. For instance, in rabbits, partial meniscectomy can be performed either on medial or lateral menisci, resulting in different outcomes. In contrast to humans and rodents, rabbits load the lateral compartment of the joint more than the medial one, thus partial lateral meniscectomy, compared with partial medial meniscectomy, results in more severe lesions and rapidly progressing OA [15].

Medial meniscal tear is achieved through transection of the MCL to expose the meniscus in the knee of an animal model. This surgical procedure induces rapidly progressing OA leading to AC degradation and joint instability similar to alterations and morphological lesions of tissues in human osteoarthritic joints upon medial meniscal tear [15].

Oestrogen deficiency in postmenopausal women has been related to osteoporosis and consequently increased risk of developing OA [100]. Thus, ovariectomised animal models can be used to study oestrogen deficiency as a potential cause of OA and determine the protective function of oestrogen. Mature ovariectomised rats develop osteoarthritic lesions of the stifle 9 weeks after surgery and have been shown to be the most representative models for the human postmenopausal OA [101]. Ovariectomised rabbits have been used to study direct effects of oestrogen insufficiency on OA development [102]. Other model organisms including mice, guinea pigs and sheep have also been used to determine the detrimental effect of oestrogen deficiency on the AC and discover new pathologic pathways. Nonetheless, the precise pathophysiological mechanism of oestrogen action is yet to be elucidated [103].

Chemically induced models of OA eliminate the need for surgery and the possibility of associated infection in some models. They are easy to induce and reproduce, and are useful for short-term studies. Administration of different dosages and intervals of compounds allows the study of various stages of disease development. These models are primarily used to study OA pain-related behaviours and the effects of drugs on major hallmarks of OA; pain and inflammation [104]. For example, in rats' intra-articular injection of MIA, a metabolic inhibitor of glyceraldehyde-3-phosphate dehydrogenase (GAPDH) activity of aerobic glycolysis pathway in chondrocytes, induces chondrocyte death, leading to osteophyte formation and AC degradation, rapid inflammation, chronic pain, hyperalgesia and allodynia [105]. The MIA rat model resembles the histological and pain-related behaviour of human OA and can therefore be more predictive of pain-alleviating drug efficacy in comparison to the other models used to test drugs for OA. However, due to rapid and widespread cell death and joint changes atypical to human primary or secondary OA pathophysiology, the validity of chemically induced models is questionable.

#### Non-invasive Models

Numerous non-invasive models of secondary OA have been described within the last few years. Each model initiates joint degeneration and produces external trauma through mechanical impact. Non-invasive mouse models of post-traumatic OA include fracture of the intra-articular tibial plateau, cyclic AC tibial compression and ACL rupture via tibial compression overload [106, 107]. These cause localised osteoarthritic joint injuries, which are not always achievable in the more invasive models, and thus allow replication of human post-traumatic OA caused by mechanical external injuries.

Fracture of the intra-articular tibial plateau is the earliest described non-invasive mouse model of secondary OA [108]. This injury model represents one of the high-energy impact injuries sustained in humans such as frontal motor vehicle collisions. In this model, the mouse knee joint was placed onto a triangular cradle of materials testing machine capable of delivering controlled loads and displacements. A wedge-shaped indenter mounted to the testing system was then used to apply compressive load to the tibia to induce articular fracture. Intra-articular tibial plateau fractures are one of the causes of post-traumatic OA in humans, therefore, this is an ideal model to study the pathological changes in the joints that occur after the acute injury.

The axial tibial loading model has been widely established in rodents and used to investigate adaptive responses of cortical and trabecular bone to mechanical loading [109–113]. In a cyclic AC tibial compression model, the external non-invasive dynamic mechanical loading is applied to the mouse tibia through the knee and ankle joints. This modifies the AC structure through a mechanoadaptive homeostatic response and contributes to OA development. One loading episode is sufficient to induce localised AC injury, whereas multiple loading episodes induce AC lesions resembling those observed in OA. The controllability of this model allows determination of the short- and long-term effects of single or multiple loading episodes on AC integrity [107]. Furthermore, intermittent non-invasive mechanical loading induced SCB thickening in these mice that may be intensified locally by adjacent AC lesions, thus indicating a spatial link between changes in SCB architecture and AC lesions following a mechanical trauma [114].

Similarly, the tibial compression model has been developed for studies of post-traumatic OA to create an acute knee injury in animals by rupturing the ACL [106]. The injury pathology of this model closely replicates ACL injury in humans. Tibial compression with a high peak force overloading regimen was sufficient to induce ACL rupture in young 8-week-old C57BL/6 mice [115]. Ruptured ACL and lax fibre alignment in the ligament led to instability of the whole joint, increased joint gap and anterior translocation of the tibia in relation to femur. Due to the change of position of bones in the knee joint the AC was no longer loaded in the same way as in the previously discussed cyclic tibial compression model, leading to the apoptosis of cells and eroded AC in multiple locations with degenerations often extending to SCB. The destabilisation of the joint upon ACL rupture, the increased concentration of inflammatory cytokines and hemarthrosis caused rapid synovial inflammation and synovial cell proliferation, leading to the formation of visible ectopic cartilaginous nodules (neocartilage metaplasia) 2 weeks post-injury [116]. Thus, this model is useful for studies of early osteoarthritic changes and the acute processes initiated by ACL rupture. Further, this model can be used to study low energy sport injuries, as well as the effect of early treatments following acute injuries. The tibial compression overload model may not be useful for long-term studies to determine the onset of OA due to severe osteophyte formation observed as part of the compensatory mechanism to joint instability.

## Conclusion

This review provides an overview of the common in vitro and in vivo models currently being used to study the pathogenesis of primary and secondary OA. 2D in vitro cell culture models offer numerous benefits and facilitate the elucidation of the molecular mechanisms underlying disease pathology. 2D models are quick, easy to work with and are financially viable; however, they have limitations including chondrocyte de-differentiation, and a reduced capacity to investigate cell–cell and cell-ECM interactions. Current 3D models provide a solution to some of these limitations; however, at present they lack biological complexity, and results generated do not often compliment results generated in animal models. There is therefore a pressing need for the development of novel, physiological, 3D cell culture models to provide alternatives to in vivo studies. The use of animal models is however an imperative stepping-stone for the preclinical discoveries, from fundamental to translational research. They are powerful research tools for studying disease pathogenesis and for developing novel targeted therapeutic interventions for OA. To date, there is no single ideal experimental model that permits investigation of all features of OA, and consideration of the advantages and disadvantages of each model is instrumental when designing a study. Another important consideration is the 3Rs (reduction, refinement and replacement), the ethical framework for conducting scientific experiments involving animals, to ensure that studies are well-designed, controlled, powered, evaluated and reported. The continuing identification and development of suitable OA models are still needed and substantial work remains before the results from these models are truly translatable to the human condition.
